# Prospective Observational Study of Microbiology of Infected Diabetic Foot Ulcers in a Tertiary-Care Hospital

**DOI:** 10.7759/cureus.71705

**Published:** 2024-10-17

**Authors:** Abhishek S Satpathy, Bhabani Patnaik, Kailash Chandra Mohapatra

**Affiliations:** 1 Surgery, SCB Medical College and Hospital, Cuttack, IND; 2 Microbiology, SCB Medical College and Hospital, Cuttack, IND

**Keywords:** antimicrobial sensitivity, culture, diabetic foot ulcers, escherchia coli, microbiology, monomicrobial, polymicrobial

## Abstract

Introduction: Diabetic foot ulcers (DFUs) are a major cause of morbidity and mortality in people with diabetes mellitus. DFUs are the leading cause of hospitalisation for diabetic patients worldwide, especially in developing countries such as India. This study presents the microbiology profile of DFUs in a tertiary care hospital in the eastern part of India.

Methodology: We conducted a prospective observational study for 150 DFU patients over a period of two years. We took samples from the DFUs of these patients, cultured them, and tested their antimicrobial susceptibility.

Results: Most diabetic foot infections (DFIs) in our study patients were caused by Gram-negative bacteria, mainly *Escherchia coli* (*E. coli*) (19.3%), *Pseudomonas* (14%), and *Staphylococcus aureus* (*S. aureus*) (12%). The majority of Gram-positive bacteria were susceptible to linezolid, followed by vancomycin and amoxycillin, while the majority of Gram-negative bacteria were susceptible to imipenem and meropenem, followed by ampicillin with sulbactam, amikacin, gentamicin, and ciprofloxacin. Our study revealed that 88.3% of the isolates were monomicrobial.

Conclusion: These findings demonstrated the importance of a local antibiogram, a microbiological exam, and antimicrobial susceptibility testing before starting antibiotic treatment for infections caused by DFUs. This protocol is different from the recommended guidelines for using empirical antibiotics.

## Introduction

A main cause of morbidity in developing nations such as India is diabetic foot infection (DFI). As a result of increased reporting of these infections as a prevalent issue among diabetics, they are now widely acknowledged as the most common diabetes-related cause of hospitalisation.

According to an Indian study, wound infections account for 30.4% of infections in diabetics [1]. A major complication of diabetes is diabetic foot ulcer (DFU). A lifetime cumulative risk of 25% is associated with foot ulcers in diabetics, with a higher susceptibility to infections in 40-80% of cases [2].

Each year, diabetes causes 80% of non-traumatic amputations, and DFUs also have a significant death and morbidity rate [3]. Over 32 million individuals in India suffer from diabetes, and it is the cause of approximately 40,000 lower limb amputations annually [3]. Osteomyelitis or soft tissue infections are two possible causes of these DFIs [3]. Important risk factors for problems in diabetics with DFIs include vasculopathy, infection frequency, peripheral neuropathy with loss of feeling, and unnoticed injuries [4]. S*taphylococcus aureus *(*S. aureus*),* Streptococcus pyogenes *(*S. pyogenes*),* Enterococcus, *and* Pseudomonas* spp. are common pathogens found in DFI [2]. The main risk factors for the development of DFUs are peripheral neuropathy, infection, abnormalities of the foot, and slight trauma to the foot. Cigarette smoking, inadequate glycaemic management, a history of foot ulcers or amputations, diabetic nephropathy, and ischemia of the minor and major blood vessels are among the additional risk factors [5]. However, by using preventive education, close monitoring, multidisciplinary treatment, organisational support, and prevention, healthcare practitioners can lower the occurrence of DFUs. In many areas, obstacles to implementation must be removed as well [6].

Simple screening instruments should be used to identify vulnerable people early on. Appropriate lifestyle modification also helps to delay or prevent the onset of diabetes, which lessens the burden on the community and the country as a whole. Healthcare professionals must use a multidisciplinary strategy to manage foot ulcers. Key components of managing DFUs are debridement, unloading, and infection control. It is also essential to manage local and systemic variables. Blood glucose control helps to reduce the chance of a recurrence, in addition to aiding in the resolution of the present wound status. It is crucial to manage underlying systemic illnesses, such as obesity, kidney disease, atherosclerotic heart disease, hypertension, and hyperlipidaemia. Additionally, addressing vascular insufficiency, providing wound care, unloading the ulcerated area, and treating infection with appropriate antibiotics are essential. Creating a moist wound bed is a crucial part of topical wound care. For improved wound healing, debridement, revision surgery on the skeletal structure, vascular reconstruction, and soft tissue covering may be necessary [5].

Appropriate systemic antibiotic therapy is necessary for the management of a clinically evident DFI, and this medication is ideally directed by determining the bacteria responsible. According to the National Institute for Health and Care Excellence (NICE) 2016 guidelines, care pathways for addressing DFIs with particular antibiotic regimens that take local resistance issues into consideration should be in place in all primary care settings. The selection of antibiotics ought to be guided by the identification of probable causal microorganisms, the intensity of the illness, and evidence of DFI efficacy while keeping expenses in mind. The NICE recommendations further suggest that the patient’s medical history, preferences, clinical context, and care setting should be considered when choosing an antibiotic [7]. For the selection of empirical antibiotic therapy, India continues to refer to the global guidelines published by the International Working Group on Diabetic Foot (IWGDF) and the Infectious Disease Society of America (IDSA). More research is required, particularly in Odisha, to determine the bacterial kind and local susceptibility pattern [8-10].

Due to a lengthy history of antibiotic usage, hospitalisation, duration, and wound grade, the bacterial pattern and antibiotic susceptibility constantly alter over time [8,11]. This study was conducted to ascertain the pattern of bacteria and the antibiotic susceptibility test on DFI in a tertiary referral hospital in Odisha. Clinicians should find the results useful in rationally using empirical antibiotics and in identifying the selection criteria.

## Materials and methods

The study conducted was a prospective cohort study with sample size of 150 patients over a period of two years at Department of General Surgery, SCB Medical College and Hospital, Cuttack. It was approved by Institutional Ethics Committee, SCB Medical College and Hospital, Cuttack, Odisha, on May 8, 2023, under expedited review with Institutional Ethics Committee application number 1370.

Inclusion criteria

It includes long-standing and newly diagnosed type 1 and type 2 diabetes with foot ulcers of Wagner grade 1 and above.

Exclusion criteria

The study does not include foot ulcers of cause other than diabetes (e.g. traumatic, foreign body, malignancy, or vasculitis), patients with DFUs who are critically ill and cannot provide consent, Wagner grade 0 DFU, patients with no bacterial growth on DFU, patients in immunocompromised state, patients with systemic malignancy, patients with end-stage renal disease, patients with decompensated liver disease, and those with decompensated cardiac disease.

Doctors conducted interviews with diabetic patients to learn more about the type, length, treatment plan, degree of glucose control, and existence or absence of chronic diabetes complications, such as a history of ulcers or lower limb amputation. The tingling, numbness, and burning sensation associated with peripheral vascular disease as well as neuropathic symptoms were assessed for both feet, which included checking for abnormalities in the nail and skin (e.g. hammer toes and claws), oedema, infection, ulceration, callus, and blistering. Amputation and gangrene were also reported. Wagner’s classification system was used to categorise ulcers (Table 1) [11]. In addition, the patients’ blood pressure was taken. Every patient’s height, weight, electrocardiogram (ECG), amount of insulin used, and length of time using insulin were documented. The hospital’s biochemistry department assessed additional clinical measures, including haemoglobin, lipid profiles, serum electrolytes, cell counts, and packed cell volume (PCV). All further diseases and problems were noted as well. For microbiological research, clinical specimens including tissue biopsies and pus samples were collected and processed. Testing for antibiotic susceptibility was executed on the clinical isolates that were taken from pus and tissue samples. All the data were collected in excel format in a coordinated endeavour by three persons, inclusive of the authors.

**Table 1:** Wagner grading of DFUs DFU: Diabetic foot ulcer

Wound grades	Description of grades
0	No lesion
1	Superficial ulcer
2	Deep ulcer
3	Abscess osteitis
4	Gangrenous forefoot
5	Gangrenous whole foot

Identification of clinical isolates

After lightly precleaning the wounds with saline, the on-duty physician took tissue samples from necrotic wounds or severed toes. The samples were then packed in sterile saline and sent to the microbiology laboratory, which was situated next to the collecting ward. After precleaning the wounds, pus samples were aspirated from deep abscesses. In other cases, swabs were collected by focusing on the moist, necrotic portions of the ulcer, which are likely to include populations of both aerobic and anaerobic microorganisms.

Clinical processing of tissue specimens

The microflora of 150 tissue samples were recorded through culture. After extracting tissue biopsies or an amputated toe, the necrotic or gangrenous portion was cut using a scalpel and forceps, put on a slide, and stained with Gram stain. On one corner of each set of media agar plates (i.e. selective and non-selective), small portions of gangrenous tissue were positioned and incubated under both aerobic and anaerobic conditions. Since the hospital does not have access to anaerobic culture techniques, patients with deep-seated abscesses that exhibit bacteria in their Gram stain and are culture-negative may receive anaerobic treatment and have their progress tracked.

Following their corresponding incubation times, the aerobes were identified by their morphological traits: size, colony colour, texture, consistency, and, most importantly, Gram staining. The catalase, nitrate, and spot indole tests were performed on the colony types to exclude the possibility of Gram-negative aerobes on the anaerobic plates. For aerobes, standard biochemical testing was done. Furthermore, on Muller-Hinton agar plates containing 4% NaCl, oxacillin discs (6 μg/ml) were utilised to identify methicillin-resistant and methicillin-sensitive *Staphylococcus *species.

Antibiotic susceptibility testing was conducted on the following antibiotics: amikacin, amoxycillin + clavulanic acid, ampicillin + sulbactam, azithromycin, aztreonam, cefadroxy, cefdinir, ceftriaxone + sulbactam, cefotaxime, cefuroxime, ciprofloxacin, ceftazidime, cefoperazone + sulbactam, cefepime, cefpirome, cefoxitin, colistin, cotrimoxazole, clindamycin, cefixime, doxycycline, tetracycline, minocycline, ertapenem, erythromycin, furazolidone, gentamicin, imipenem, linezolid, levofloxacin, meropenem, moxifloxacin, nalidixic acid, norfloxacin, netilmycine, nitrofuantion, ofloxacin, penicillin, piperacillin + tazobactam, polymixin - B, roxithromycin, tigecycline, ticarcillin + clavulanic acid, vancomycin, cefpodoxime, tobramycin, daptomycin, and teicoplanin. All of the antibiotics had minimum inhibitory concentration (MIC) values between 0.01 and 256 μg/mL. Aerobes were routinely tested for antibiotic sensitivity using the Kirby Bauer’s disc diffusion method. In the case of aerobes, the zone of inhibition surrounding the tested antibiotics was evaluated, and conclusions were drawn using the breakpoints. Data collected from the questionnaire and the culture-sensitivity report were then analysed using SPSS version 22, and the results were recorded.

## Results

The mean age of the study participants was 55.11 ± 15.78 years, with a minimum age of 21 and a maximum of 90 years. Age category-wise distribution suggests that the majority belonged to age between 40 and 60 years (42%) followed by more than 60 years (38%). Most study participants were male (64%) than female (36%).

**Table 2:** Socio-demographic characteristics of the study population (N=150)

Characteristics		Frequency (Percentage)
Age category	< 40 years	30 (20%)
	40-60 years	63 (42%)
	> 60 years	57 (38%)
Gender	Male	96 (64%)
	Female	54 (36%)
Locality	Rural	86 (57.3%)
	Urban	64 (42.7%)
Duration of diabetes	< 10 years	98 (65.3%)
	≥ 10 years	52 (34.7%)

Most of the study population belonged to rural areas (57%). The mean duration of diabetes was 10.53 ± 8.34 years, with a minimum of 1 year to a maximum of 38 years. Almost two-thirds of the participants (65%) had diabetes for less than 10 years. Details are given in Table 2.

**Table 3:** Wagner’s grading of the DFU (N=150) DFU: Diabetic foot ulcer

Grading	Frequency (Percentage)
I	27 (18%)
II	58 (38.7%)
III	42 (28%)
IV	16 (10.7%)
V	7 (4.7%)

Table 3 shows Wagner’s grading of DFUs. The majority of the patients had grade II ulcers (38.7%), followed by grade III (28%) and grade I (18%). Only a few have grade V ulcers (4.7%).

**Table 4:** Culture results of the DFU patients (N=150) DFU: Diabetic foot ulcer

Microbes	Frequency (Percentage)
None	9 (6%)
E. coli	29 (19.3%)
Pseudomonas	21 (14%)
S. aureus	18 (12%)
Klebsiella	18 (12%)
Polymicrobes	17 (11.3%)
Acinetobacter	13 (8.7%)
P mirabilis	7 (4.7%)
Enterococcus	7 (4.7%)
Coagulase -ve staph	4 (2.7%)
K. oxytoca	5 (3.3%)
Diphtheroid	2 (1.3%)

Table 4 shows the culture results among the study population. Nine percent of the patients were culture negative, and the rest were culture positive (91%). Major bacteria identified in the ulcers were *E. coli *(19.3%), *Pseudomonas* (14%), and *S. aureus* (12%).

**Table 5:** Gram stain results among the bacteria isolated from study participants

Gram Stain	Frequency (Percentage)
Gram +ve	33 (22%)
Gram -ve	117 (78%)

Majority of the ulcer showed no more than one bacteria in the culture results (82.7%) while 11.3% had polymicrobial isolates and 6% were culture negative. However, majority of the polymicrobial culture were associated with *Pseudomonas aeruginosa* (Tables 4, 6, 7).

**Table 6:** Polymicrobial culture associated with Pseudomonas aeruginosa among patients

Microbes associated with *Pseudomonas*	Frequency (Percentage; out of 150)
Enterococcus	2 (1.3%)
Klebsiella pneumoniae	8 (5.4%)
E. coli	1 (0.7%)
Klebsiella oxytoca	1 (0.7%)
Proteus mirabilis	2 (1.3%)

**Table 7:** Other polymicrobial culture results

Polymicrobial culture	Frequency (Percentage; out of 150)
*Acinetobacter *and* Klebsiella*	1 (0.7%)
*E. coli *and* Acinetobacter*	1 (0.7%)
*E. coli *and* Proteus mirabilis*	1 (0.7%)

Drug sensitivity results are shown in Tables 8, 9.

Majority of Gram-positive bacteria were susceptible to linezolid followed by vancomycin and amoxycillin.

Majority of Gram-negative bacteria were susceptible to imipenem and meropenem, followed by ampicillin with sulfbactam, amikacin, gentamicin and ciprofloxacin.

**Table 8:** Drug sensitivity among Gram-positive bacteria (N=150)

Drugs	Frequency (Percentage)
Amoxycillin	3 (2%)
Linezolid	12 (8%)
Vancomycin	6 (4%)
Cefotaxime	1 (0.7%)
Ceftriaxone	2 (1.3%)
Cloxacillin	1 (0.7%)
Imipenem	1 (0.7%)
Meropenem	1 (0.7%)

**Table 9:** Drug sensitivity among Gram-negative bacteria (N=150)

Drugs	Frequency (Percentage)
Amikacin	7 (4.7%)
Ampicillin with sulbactam	9 (6%)
Azithromycin	1 (0.7%)
Cefepime	2 (1.3%)
Cefotaxime	2 (1.3%)
Cefoxitin	1 (0.7%)
Ceftriaxone	4 (3.7%)
Ciprofloxacin	7 (4.7%)
Clarithromycin	2 (1.3%)
Ertapenem	2 (1.3%)
Gentamicin	7 (4.7%)
Imipenem	12 (8%)
Levofloxacin	6 (4%)
Amoxycillin	3 (2%)
Meropenem	11 (7.7%)

The drug susceptibility profile is shown in Table 10 describes polymicrobial culture results with common drug susceptibility profiles.

**Table 10:** Common antimicrobials susceptible to polymicrobial resistance bacteria

Drugs	Frequency (Percentage)
Ampicillin with sulfbactam	1 (0.7%)
Capreomycin	2 (1.3%)
Cefotaxime	1 (0.7%)
Ceftriaxone	1 (0.7%)
Cotrimoxazole	2 (1.3%)
Ertapenem	1 (0.7%)
Gentamicin	2 (1.3%)
Imipenem	2 (1.3%)
Levofloxacin	2 (1.3%)
Piperacillin with tazobactam	2 (1.3%)
Polymyxin B	1 (0.7%)

**Figure 1:**
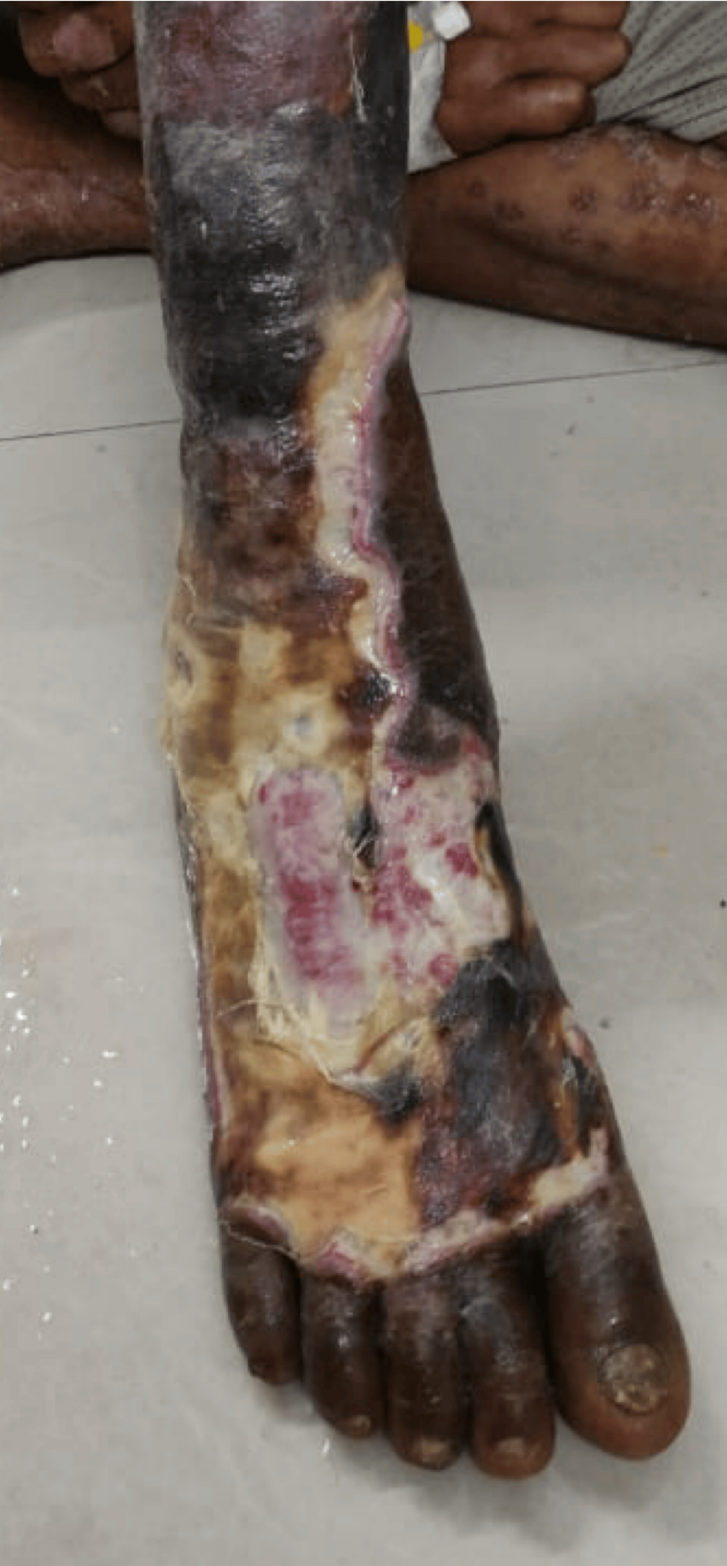
Typical DFI before debridement from which infected tendon was taken for culture DFI: Diabetic foot infection

**Figure 2:**
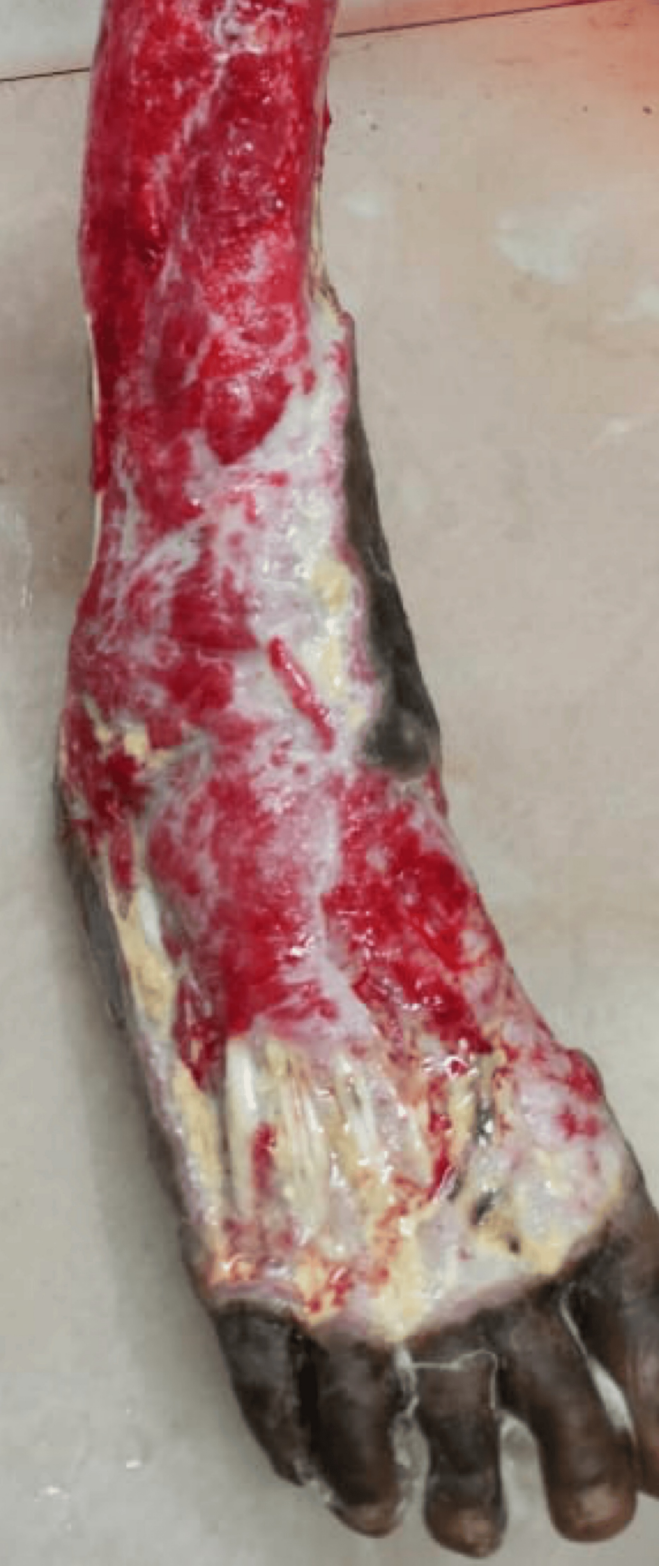
DFI after debridement DFI: Diabetic foot infection

The majority of DFU patients underwent debridement (48%), followed by debridement with ray amputation (20%) and conservative treatment (14%) (Table 11). Only five patients had either knee- or ankle-level amputation. An instance of successful debridement is illustrated in Figure 1 (pre-debridement) and Figure 2 (post-debridement).

**Table 11:** Type of management among the patients (N=150)

Management	Frequency (Percentage)
Conservative	21 (14%)
Debridement	72 (48%)
Debridement with fasciotomy	11 (7.3%)
Debridement with grafting	11 (7.3%)
Debridement with ray amputation	30 (20%)
Amputation	5 (3.3%)

In Table 12, we compared the socio-demographic factors and other clinical factors with the culture results. Most commonly, males had positive culture results. Positive culture results were also seen in patients in the 40-to-60-years age group. Most patients from rural areas had culture-positive results. Patients with diabetes duration of less than 10 years had a higher proportion of culture-positive results in our study. Moreover, Wagner type II was predominantly associated with culture-positive results.

**Table 12:** Association of different risk factors with culture results (N=150)

Properties		Culture positive N (%)	Culture negative N (%)
Gender	Male	89 (63.1)	7 (77.8)
	Female	52 (36.9)	2 (22.2)
Age	< 40 years	29 (20.6)	1 (11.1)
	40-60 years	56 (39.7)	7 (77.8)
	> 60 years	56 (39.7)	1 (11.1)
Locality	Rural	80 (56.7)	6 (66.7)
	Urban	61 (43.3)	3 (33.3)
Duration of diabetes	< 10 years	93 (66.0)	5 (55.6)
	≥ 10 years	48 (34.0)	4 (44.4)
Wagner’s grading	I	26 (18.4)	1 (11.1)
	II	55 (39.0)	3 (33.3)
	III	38 (27.0)	4 (44.4)
	IV	15 (10.6)	1 (11.1)
	V	7 (5.0)	0 (0)

## Discussion

The study population’s mean age fell into the 40-60 years age range (42%) since these individuals tend to be the most active, which impacts their feet. Studies by Zhang et al. and Rossboth et al. indicate that DFUs are more common in 40-year-olds and > 60-year-olds [12,13]. Males comprised the majority of patients with DFIs, and most of them came from rural locations. In their studies, James et al. and Rossboth et al. found similar demographics [12,14]. The prevalence of men in DFU may be connected to, for example, gender-specific lifestyle characteristics and occupations that put strain on the feet. Males were more likely than females to work outside more frequently and to not take proper care of their feet.

Wagner grades I through V were applied to the wounds that were reported in our study. Grade II ulcers accounted for 38.7% of the patients, grade III ulcers for 28%, and grade I ulcers for 18%. Just 4.7% of people have grade V ulcers. A similar study by Rooh-ul-Muqim evaluated DFUs with Wagner grading and showed that majority of diabetic foot lesions were in grade 2 to 5 with lesser grade lesions simply treated by conservative approach and higher grade lesions needing surgical debridement and amputations [15].

In our study, 91% of the patients had positive cultures, while just 9% had negative cultures. The three main bacteria found in the ulcers were *S. aureus *(12%), *Pseudomonas *(14%) and* E. coli* (19.3%). Anaerobic, picky bacteria, mycobacteria, or any fungal invasion are indicated by a negative culture. In contrast to Spichler et al.'s findings, which showed that the majority of DFIs were polymicrobial, the majority of infections in our sample were monomicrobial [16]. Only 11.3% of the ulcers exhibited polymicrobial isolates, whereas the majority (88.7%) had only one microbe detected in the culture findings. Furthermore, *S. aureus* was the most often identified Gram-positive bacterium from individuals with diabetic foot disease in our investigation. This is comparable to the findings of Afonso et al., who identified primarily monomicrobial Gram-negative bacteria from their patients with DFUs [17]. Monomicrobial infection was found to be the major cause of DFUs in individuals with comparable conditions, according to a related study conducted by Seth et al. [18].

Moreover, a research by Li et al. on the organisms causing diabetic necrotizing fasciitis revealed that a predominant Gram-negative monomicrobial infection was involved [19].

Most DFIs in our study were treatable with more recent antibiotics (e.g. imipenem and linezolid). Most isolated Gram-positive organisms were susceptible to linezolid and vancomycin, but most isolated Gram-negative organisms were susceptible to imipenem, meropenem, amikacin, gentamicin, and ciprofloxacin. Similar to studies conducted by Du et al. on the microbial distribution among patients with DFUs in China, the majority of infections were monomicrobial, with Gram-negative infections being the most prevalent and mostly sensitive to piperacillin/tazobactam, amikacin, meropenem, and imipenem. Most of the Gram-positive bacteria found in the study were susceptible to teicoplanin, vancomycin, and linezolid [20]. Similar research by Mashaly et al. on the isolation of aerobic bacteria from DFIs in Egypt revealed that the most effective antibiotics for Gram-negative bacilli were amikacin, tigecycline, and meropenem, while for staphylococci they were linezolid and vancomycin [21]. Huwae et al. reported a study in which *Pseudomonas aeruginosa *and* S. aureus* were the most frequently isolated aerobic microorganisms from DFIs from Asia between 2018 and 2022. Gram-positive bacteria have shown effective sensitivity to vancomycin, linezolid, doxycycline, chloramphenicol, and trimethoprim-sulfamethoxazole. Gram-negative bacteria demonstrate excellent susceptibility to aminoglycosides, carbapenems, and piperacillin-tazobactam [22]. In a tertiary-care hospital in Egypt, a study by Ismail et al. on DFIs revealed the prevalence of major monomicrobials, particularly *Pseudomonas* and *Proteus* species. Additionally, this investigation demonstrated that the most effective antimicrobial medications against Gram-positive bacteria were vancomycin, teicoplanin, and linezolid, while the most effective antimicrobial agents against Gram-negative pathogens were colistin, imipenem, meropenem, and piperacillin-tazobactam [23].

In our study, debridement was performed on 48% of patients with DFUs, followed by conservative treatment (14%), debridement with ray amputation (20%), and debridement alone. Amputations at the ankle or knee levels were performed on just five patients. This is comparable to the research by Piagessi et al. that demonstrated the superiority of surgical care over the conservative method for DFIs [24]. Similar research by Aamir et al. described the treatment of DFIs at tertiary care hospitals, with superficial debridement and toe and foot amputation being the most frequently performed surgical procedures [25].

In our investigation, patients from rural areas (88.2%) and females (58.8%) accounted for the majority of polymicrobial isolates. Numerous factors, including, cultural diversity, geographical variations, awareness, and antibacterial treatment, were found to be involved in the pathophysiology of DFIs, particularly polymicrobial infections, according to a study conducted by Kale et al. on the bacterial diversity in DFIs in India [26].

In our study, the majority of DFIs came from rural locations. It matched the findings of a study by Viswanathan et al. that examined DFIs in rural and urban settings. In comparison to patients in cities, he found that foot ulcers were more common in rural areas. Rural patients were more likely to experience reulceration and require surgical intervention, even when they received counseling comparable to that of urban patients [27].

**Table 13:** Represents studies done regarding DFIs with their culture results MRSA: Methicillin-resistant *Staphylococcus aureus*; DFI: Diabetic foot infection

Authors	Aims of the study	Sample, methods	Key findings
Macdonald et al. [28]	Evaluating microbiology of DFIs	Meta-analysis of 112 studies representing 16,159 patients	Most common organism was *S. aureus*, of which 18% was MRSA, with other common organisms being *Pseudomonas, Enterococci *and* E. coli.*
Palomo et al. [29]	Evaluating microbiology of DFIs in a tertiary-care hospital in Sao Paulo, Brazil	Retrospective cohort of 320 patients	Most common organism was *Enterococcus faecalis* followed by *S. aureus* and coagulase negative staphylococci with most common among Gram negative being *Pseudomonas aeruginosa*.
Kow et al. [30]	Evaluating microbiology of DFIs in three district hospitals of Malaysia	Retrospective cohort of 173 patients	Most common organism isolated was *S. aureus* followed by *Klebsiella, Pseudomonas *and* Proteus* spp. Gram-positive pathogens were sensitive to most of the antibiotics tested except penicillin and fusidic acid. Gram-negative pathogens were sensitive to all antibiotics tested except ampicillin and amoxicillin/clavulanic acid. Amikacin provide coverage for all Gram-negative pathogens in DFI.
Kale et al. [26]	Evaluating etiological trends and bacterial diversity in Indian DFIs	Meta analysis of 56 articles from 2005 to 2022	Gram-negative pathogens more common than Gram-positive pathogens. Most common among Gram-negative were *E. coli* followed by *Pseudomonas aeruginosa, Klebsiella *spp.and* Proteus *spp. Among Gram-positive were *S. aureus *followed by* Enterococcus *spp.

 Limitations of the study

Due to unavailability of anaerobic and fungal culture methods, these bacteria can’t be isolated and hence could not be represented in our study. Also, as small sample size had (convenient sampling) been taken, the findings could not be perfectly extrapolated to the entire population. Certain bacteria isolated may be contaminants or colonisers and may not be actively involved in pathophysiology of DFI. Furthermore, biopsy material not reaching on time may have affected culture results.

## Conclusions

A main cause of morbidity in developing nations such as India is DFI. Choosing proper antibiotics is crucial when planning treatment for DFIs. Empirical antibiotic therapy based on susceptibility data obtained from our study is the mainstay of the condition’s early care. It is essential to comprehend the underlying cause of DFIs to select the appropriate medications and to effectively treat these infections. After conducting a comprehensive clinical and microbiological evaluation, our study generated an antibiogram based on data from antibiotic susceptibility patterns in patients with DFIs in a tertiary care teaching hospital. It thus conveyed the importance of a local antibiogram, the need for a microbiological examination, and the requirement for antimicrobial susceptibility testing prior to initiating antibiotic treatment for infections resulting from DFUs, in contrast to the suggested international guidelines for empirical antibiotics.

## Figures and Tables

**Figure 3:**
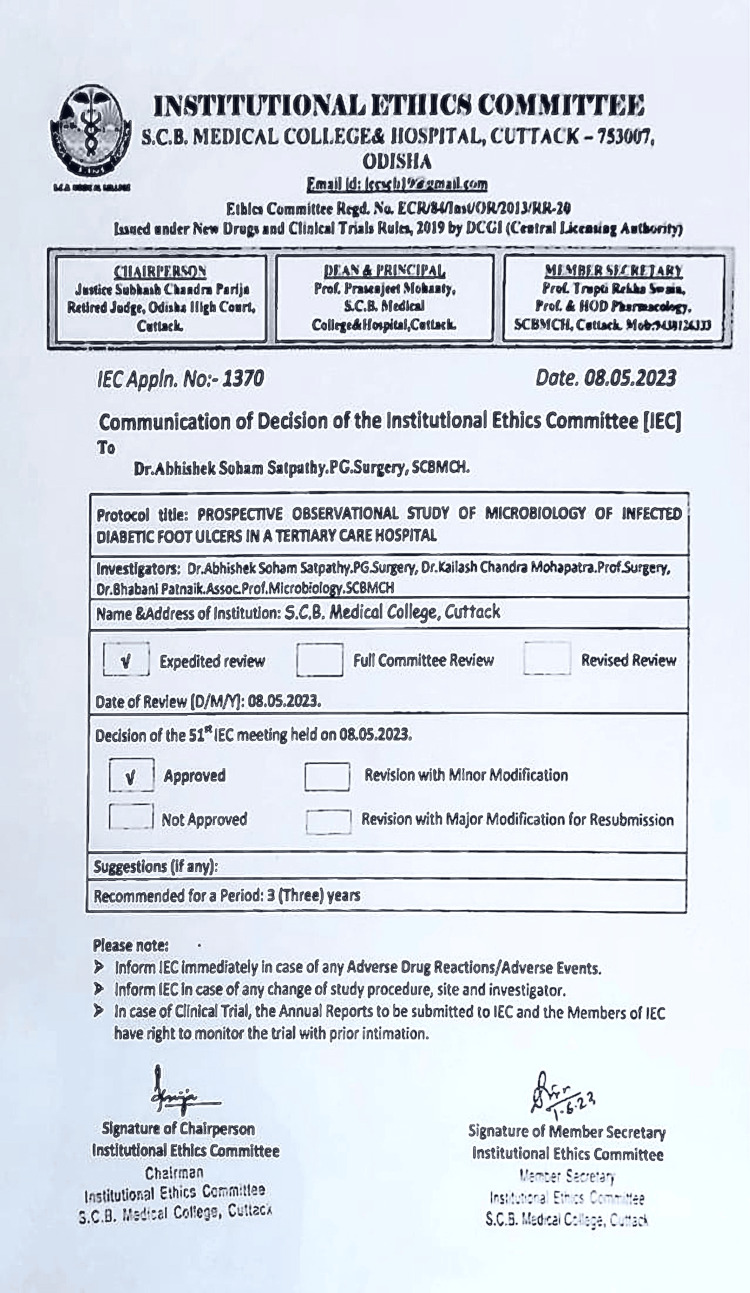
IRB approval of the study
